# Destroying *c-jun* Messenger: New Insights into Biological Mechanisms of DNAzyme Function

**DOI:** 10.18632/oncotarget.549

**Published:** 2012-07-13

**Authors:** Levon M. Khachigian, Hong Cai, Fergal J. Moloney, Christopher R. Parish, Beng H. Chong, Roland Stocker, Ross St.C. Barnetson, Gary M. Halliday

**Affiliations:** Centre for Vascular Research, University of New South Wales, Sydney, Australia; Centre for Vascular Research, University of New South Wales, Sydney, Australia; Dermatology Research Laboratories, University of Sydney, Australia and Dermatology Department, Mater Misericordiae University Hospital, Dublin, Ireland; Centre for Vascular Research, John Curtin School of Medical Research, Canberra, Australia; Centre for Vascular Research, University of New South Wales, Sydney, Australia; Centre for Vascular Research, University of Sydney, Sydney NSW Australia and Victor Chang Cardiac Research Institute, Sydney, Australia; Dermatology Research Laboratories, University of Sydney, Australia; Dermatology Research Laboratories, University of Sydney, Australia

Skin cancer is the commonest cancer in light-skinned Caucasians and non-melanoma skin cancer accounts for over 90% of these malignancies [1]. Basal cell carcinoma (BCC) generally do not metastasize, but can be locally invasive. Squamous cell carcinomas (SCC) typically occur on chronically sun-exposed sites such as face and forearms and are at increased risk of metastatic spread particularly in immunosuppressed individuals. Surgery, the principal treatment for these non-melanoma skin cancers, can cause disfiguring scars while other therapeutic options are limited by side effects or lack of efficacy.

Work by our group and others has demonstrated that immediate-early genes can serve as key targets in a range of cancer types. The c-*jun* gene is mapped to 1p32-p31 and encodes the 45kDa bZIP-domain-containing transcription factor c-Jun that, in combination with protein partners, forms AP-1. Protein partners of c-Jun are many and include c-Fos, pRb, BRCA1, ATF-2 and ERG. c-Jun/AP-1 is dynamically regulated by growth factors and cytokines, is overexpressed in a range of cancers including BCC, SCC and melanoma, and stimulates the expression of numerous genes [2-4].

DNAzymes are single-stranded synthetic DNA-based catalytic molecules that can be engineered to bind and destroy target messenger RNA [5]. These agents have been used as inhibitors of biological processes in a range of animal models of human disease including ocular neovascularization, kidney disease and spinal cord injury [6]. The first *in vivo* demonstration of efficacy was the use of DNAzymes targeting the transcription factor early growth response (Egr)-1 as inhibitors of intimal thickening in a rat model of balloon angioplasty [7]. The wider use of DNAzymes as therapeutic agents has been hampered by delivery issues, particularly the limitation in target tissue delivery associated with systemic administration [8]. This notwithstanding, it has been suggested that oligonucleotides *in vivo* do not necessarily require a delivery vehicle for endosomal/lysosomal sequestration [9].

Our recent work with local administration of liposomal formulation of c-Jun-targeting DNAzymes (Dz13) [2] has overcome some of these systemic delivery issues. Dz13 inhibited human BCC growing as intradermal tumors in SCID mice, and blocked the growth of SCC as intradermal and subcutaneous implants [2, 3]. Inhibition of tumor growth was Dz13 dose-dependent and sustained. At the highest dose used, Dz13-treated BCC did not regrow even 3 weeks after the cessation of treatment. A control DNAzyme with scrambled binding arms or single point mutation in the catalytic domain did not affect tumor growth. Dz13 inhibited the expression of c-Jun *in vivo*, demonstrating that it acted on its target, and this resulted in a decrease in CD31^+^ staining in the tumors, an indicator of tumor angiogenesis [2]. In addition, Dz13 reduced lung nodule formation in a model of SCC metastasis.

The study by Cai and co-workers provided novel insights into the mechanism of action of DNAzymes [2]. Dz13 rendered c-*jun* mRNA unstable, reduced growth factor expression and increased apoptosis in the tumors without apparent induction of oxidative stress. Interestingly, Dz13-mediated tumor decay was more profound in immunocompetent mice syngeneic to the tumor compared with immunocompromised animals. Immunohistological inspection revealed increased immune and inflammatory cells in Dz13-treated tumors in the immunocompetent mice. In addition, Dz13 mediated tumor regression was prevented by the administration of CD4 or CD8 antibodies, which depleted the mice of the respective T cell subsets. Thus, inhibition of tumor growth by a DNAzyme involves the induction of tumor immunity. These findings suggest that c-Jun inhibition in tumors stimulates apoptosis and adaptive immune mechanisms that attack the tumor. Underpinned by a favorable preclinical safety profile, DNAzymes could provide a new treatment option combining both direct and indirect mechanisms to prevent the growth and spread of non-melanoma skin cancer.
